# La grossesse chez les hémodialysées chroniques

**DOI:** 10.11604/pamj.2015.20.213.6196

**Published:** 2015-03-10

**Authors:** Bouchra Doukkali, Abdelaali Bahadi, Hicham Rafik, Driss Kabbaj, Mohamed Benyahia

**Affiliations:** 1 Hôpital Militaire d'Instruction Mohamed V, Rabat, Maroc

**Keywords:** Insuffisance rénale chronique, hémodialyse, grossesse, Chronic renal failure, hemodialysis, pregnancy

## Abstract

La survenue d'une grossesse en hémodialyse chronique (HDC) est rare, mais depuis la description du premier cas par Confortini en 1971, plusieurs observations ont été rapportées. L'hémodialyse a considérablement amélioré la fertilité de ces patientes. Nous rapportons l'expérience de douze grossesses survenues entre 1999 et 2014, chez douze patientes d’âge médian 34 ans (22-44), en hémodialyse (HD) depuis 40 mois (3-72), l’âge gestationnel moyen de diagnostic est de 16 semaines d'aménorrhée, la grossesse était compliquée dans 50% des cas par un hydramnios. Le terme moyen est de 35 semaine d'aménorrhée (SA) et l'accouchement a été réalisé dans 90% des grossesses par voie basse. Le poids moyen des nouveau-nés est de 1800g. De telles grossesses sont à haut risque du fait de la fréquence des complications. Elles devraient être contrôlées par les équipes multidisciplinaires, et la consultation prénatal ne devrait pas être négligée. L'objectif de ce travail est de rapporter notre expérience concernant la survenue d'une grossesse chez les patientes dialysées et de la confronter aux données de la littérature.

## Introduction

La survenue d'une grossesse en hémodialyse chronique (HDC) est rare, et leurs évolution est précaire mais depuis la description du premier cas par Confortini en 1971, plusieurs observations ont été rapportées [1]. En raison d'une perturbation de l'axe hypothalamo-hypophysaire, la fertilité est diminuée chez les patientes en HDC qui présentent une diminution de la libido [2], une dysménorrhée et une anovulation dans 50% des cas [3]. Selon la littérature, la fréquence de conception varie selon les pays; de 1,4% par an en Arabie saoudite [4] à 0,5% aux états unis [5] et le taux de réussite varie selon les séries de 30% [6] à 80% [7]. Ces grossesses constituent un groupe à haut risque et n'aboutissent à des nouveau-nés vivants que seulement dans 60% des cas, du fait de la fréquence des complications [8, 9]: la mère est exposée aux accidents hémorragiques dont l'hématome rétro-placentaire (HRP), à une aggravation de l'anémie, à une thrombose de l'abord vasculaire ou du circuit extracorporel et à des anomalies hépatiques dont la cholestase gravidique; le fœtus subit l'anémie maternelle et souffre d'hypoxie chronique; il est menacé d'hydramnios en cas de mauvais contrôle de la volémie; le retard de croissance intra-utérin et la prématurité sont habituels. L'objectif de ce travail est de rapporter notre expérience concernant la survenue d'une grossesse chez les patientes dialysées et de la confronter aux données de la littérature.

## Méthodes

Il s'agit d'une étude rétrospective réalisée à l'hôpital militaire d'Instruction Mohammed V (HMIMV) intéressant 12 grossesses chez 12 patientes en hémodialyse chronique colligées depuis juillet 1999 jusqu'au décembre 2014. Au cours de ces grossesses nous avons étudiés l’âge des patientes, la néphropathie initiale, date de début de la dialyse, le moyen et l’âge de diagnostic de la grossesse, le nombre et la durée des séances d'hémodialyse, les traitements reçus visant à contrôler l'anémie et la tension artérielle, les complications obstétricales, l’âge du terme, le mode d'accouchement et le poids à la naissance. L'hémodialyse a été effectuée en utilisant des membranes en polysulfone, les patientes ont été suivies en collaboration entre les obstétriciens et les néphrologues, le programme d'hémodialyse a été augmenté de 12h à 18h hebdomadaires en utilisant un débit de pompe à 250 ml/min, un bain de bicarbonate, Les évaluations de la quantité d'ultrafiltrations étaient basé sur la surveillance de la tension artérielle maternelle avant, pendant, et après la dialyse. Pour corriger l'anémie nous avons visé une hémoglobine au-dessus de 9g/dl. La croissance fœtale a été surveillée par une échographie obstétricale chaque semaine.

## Résultats

Sur la période de l’étude, nous avons dénombré 12 grossesses survenues chez 12 patientes différentes avec un taux moyen de réussite de 66,66%, soit 8 nouveaux nés vivants. Toutes bénéficiaient de l'hémodialyse périodique; nous n'avons recensé aucune grossesse en dialyse péritonéale. Aucune n’était diabétique au moment de la conception. Aucune ne prenait de contraception, ni dispositif intra utérin. Il a été difficile de retrouver dans les dossiers des informations concernant leurs cycles menstruels. L’âge moyen des patientes au moment de la conception en dialyse est de 34 ans avec des extrêmes entre 22 et 44 ans. La néphropathie est indéterminée dans 6 cas, une glomérulonéphrite chronique dans 1 cas, une néphropathie tubulo-interstitielle chronique dans 2 cas et une néphropathie vasculaire dans 1 cas. La durée moyenne d'hémodialyse avant la conception est de 40 mois avec des extrêmes entre 3 et 72 mois à raison de 3 séances de 4h chaque semaine. Cinq patientes avaient une diurèse résiduelle de plus d'un litre par 24h et le reste des patientes étaient anuriques. L’âge moyen de diagnostic de la grossesse est de 16 semaines d'aménorrhée (SA) avec des extrêmes entre 7 et 27 semaines. Le moyen de diagnostic de grossesse est par les BétaHCG dans 4 cas et dans 7 cas par échographie tandis qu'une grossesse a été diagnostiquée fortuitement lors d'une consultation pour des métrorragies (Tableau 1).

**Tableau 1:** Les caractéristiques des patientes

Nombre des grossesses	n = 12	
Age des patientes (moyen, extrêmes)	34 ans	(22-44)
Age moyen des grossesses	16 semaines	
Durée moyenne d'hémodialyse	40 mois	(3-72)
Néphropathies		
Indéterminée	6	
Glomérulopathies chronique	1	
Tubulo-interstitielle chronique	2	
Vasculaire	1	
Lithiasique	1	
Lupus	1	

Le nombre de séances de dialyse a été augmenté dès le diagnostic de grossesse à 4 fois par semaine dans cinq cas et à 6 fois dans les autres cas, dans le but de limiter les prises de poids inter-dialytiques trop importantes, et donc l'ultrafiltration excessive à chaque séance. La durée des séances a été majorée à 6h dans deux cas. Ceci correspond à une durée totale ente 12 et 24h par semaine. Toutes ont été dialysés par une fistule artério-veineuse, sans complication notable au niveau de cet abord: pas de rupture, ni thrombose, ni infection. Toutes les patientes ont bénéficié d'un bain tamponné aux bicarbonates, les débits suivants ont été observés: pompe à sang de 200 à 250 ml/min, débit du dialysat de 300 à 500 ml/min avec une anticoagulation par l'héparine de bas poids moléculaire (HBPM) et des membranes biocompatibles en polysulfone. Le poids de fin de dialyse a été ajusté sur des critères cliniques habituels: crampes, chute de la pression artérielle, et sur des échographies obstétricales réalisées à la fin des séances d'hémodialyse. L'anémie était présente dans tous les cas au moment de diagnostic de la grossesse avec un taux moyen d'hémoglobine à 8 g/dl. Pour améliorer ces chiffres on a augmenté les doses de l’érythropoïétine (EPO) dans 4 cas avec recours à la transfusion dans 7 cas et supplémentation en fer dans quatre cas. L'hypertension artérielle (HTA) est présente chez cinq patientes et traitée par un inhibiteur calcique ou un antihypertenseur central ou l'association des deux. La grossesse a été compliquée dans la moitié cas d'un hydramnios nécessitant chez deux patientes le recours à une amniocentèse. Les complications de la grossesse sont récapitulées dans le Tableau 2. L'accouchement a eu lieu par césarienne dans 2 cas, et par voie basse dans les autres cas. Sur les 12 grossesses recensés il y a eu 8 nouveaux nés vivants avec un âge du terme moyen à 35 SA et un poids moyen de 1800g (350-2900g). Aucune complication lors de l'accouchement n'a été rapportée, même pas d'incidents hémorragiques malgré l'administration d'HBPM lors des séances proches de la naissance.

**Tableau 2:** Les complications de la grossesse

	n = 12	
HTA	5	(41.66%)
RCIU	1	(8.3%)
Hydramnios	6	(50%)
Hémorragie du post partum	0	(0%)
RPM	1	(8.3%)

HTA: Hypertension artérielle, RCIU: Retard de croissance intra-utérin

RPM: Rupture prématurée des membranes

## Discussion

Le premier succès relaté date de 1971. Les grossesses en dialyse sont restées encore extrêmement rares jusqu’à la généralisation de l'utilisation de l’érythropoïétine recombinante humaine à la fin des années 80. Cette dernière, en corrigeant l'anémie, restaure une meilleure oxygénation tissulaire permettant le retour à une homéostasie correcte, avec un meilleur fonctionnement de l'axe hypothalamo-hypophysaire, et une baisse du taux de prolactine mis en cause dans les infertilités des patientes en insuffisance rénale chronique. Le taux de réussite des grossesses chez l'hémodialysée chronique varie selon les auteurs mais aussi selon les pays mais il semble aussi s'améliorer à travers les années avec des taux de succès arrivant jusqu’à 100% profitant ainsi des progrès des techniques d'hémodialyse. Dans notre série, ce taux reste diminué et ne dépassant pas les 66,6%. Ceci peut être expliqué par le caractère multidisciplinaire de la prise en charge de ces patientes en particulier néonatale qui reste déficitaire dans notre formation. Ces patientes ne bénéficient pas, en général, d'une contraception et d'un suivi gynécologique adaptés, avec une majorité de grossesses non désirées. L'absence de prévention chez ces patientes en hémodialyse chronique en âge de procréer a déjà été rapportée dans la littérature et souligne l'importance du suivi gynécologique systématique [3]. En cas de désir de grossesse, il convient d'arrêter les médicaments potentiellement tératogènes qui sont essentiellement représentés par les inhibiteurs du système rénine-angiotensine [10]. Dans littérature la durée moyenne de la dialyse est de 7 ans, dans notre série Cette prépondérance est en moyenne de 29,6 mois. Chez les femmes en âge de procréer en dialyse, en raison de l'irrégularité des menstruations, le diagnostic de grossesse est souvent retardé. Dans notre étude, le temps moyen du diagnostic était comme dans la littérature de 16 semaines [11].

Lorsque la grossesse est connue, le néphrologue a pour rôle d'adapter le protocole d'hémodialyse, d'assurer un bon contrôle de la tension artérielle, du poids sec, du taux d'hémoglobine et des apports nutritionnels: la fréquence d'hémodialyse doit être augmentée dès que possible à quatre séances puis à six séances par semaine à partir du cinquième mois pour optimiser le contrôle métabolique et de la volémie avec un objectif de 16 à 24 heures d'hémodialyse hebdomadaires (HD) qui est retrouvé associé à un meilleur pronostic fœtal [12]. Dans notre expérience, la fréquence et la durée d'HD hebdomadaire étaient modifiées, en augmentant d'abord la fréquence des séances puis le temps d'HD hebdomadaire [4, 5, 10, 13]. Une modification des paramètres de dialyse est effectuée par de nombreuses équipes néphrologiques mais pas par toutes. On peut modifier la composition du bain de dialyse en diminuant la concentration en calcium et parfois en bicarbonates, et en augmentant celle en potassium. Les débits de la pompe à sang sont de 250 à 300 ml/min dans la littérature et de 200 à 250 ml/min dans notre étude. Quant au débit du dialysat, la bibliographie annoncent des valeurs de 500 à 600 ml/min et de 300 à 500 ml/min dans notre étude. Dans notre étude. Le traitement anticoagulant perdialytique n’était pas compliqué mais en péripartum, l'utilisation doit être limitée en raison des risques hémorragiques [9]. L'adaptation régulière du poids de base est absolument nécessaire. Le plus souvent, elle suit empiriquement la prise de poids gravidique habituellement constatée chez les femmes enceintes qui est de l'ordre de + 0,5 kg par semaine ou par quinzaine environ et idéalement de 10 à 15 kg dans la littérature [14]. Dans notre série la prise de poids par semaine n'a pas été mentionnée dans les dossiers. II faudra éviter au maximum les chutes tensionnelles durant les séances qui sont très préjudiciables au fœtus, et surveiller la survenue fréquente de contractions utérines en fin des séances aux deuxième et troisième trimestres. Dans notre expérience, la fréquence de l'HTA est de l'ordre de 41,66% contre 79% pour Okundaye et coll., avec une moindre utilisation de traitement antihypertenseur et une bonne réponse à l'augmentation de la durée d'HD [5]. Un bon contrôle de la tension artérielle nécessite une évaluation juste du poids sec, estimation qui reste surtout clinique, aidée par la connaissance de la prise de poids physiologique « idéale » pendant la grossesse qui est de 1 à 1,5 kg le premier trimestre puis 500 g/semaine au deuxième et troisième trimestres [14]. L'objectif d'hémoglobine chez la femme enceinte saine est de 9 à 11,5 g/dl [15]. La transfusion doit être évitée car source d'allo-immunisation en particulier chez la femme enceinte [16]. Concernant l'utilisation d'EPO pendant la grossesse, il n'est pas retrouvé de récepteur à l'EPO dans le placenta humain ni de complications secondaires à son utilisation [5, 10, 17]. Sa posologie doit être augmentée en raison de l'augmentation des besoins en production de globules rouges [8]. L'utilisation ou l'augmentation précoce de la posologie d'EPO permet de diminuer la fréquence de transfusion de 77 à 26% [5]. Dans notre expérience, 7 patientes ont nécessité une transfusion en l'absence d'augmentation précoce de la dose d'EPO. Un problème propre aux femmes en HD est le développement de la thrombose de leurs fistules artérioveineuses, empêchant l'accès vasculaire. Dans notre série cette complication n'a été observée chez aucune patiente.

Malgré un régime de dialyse rénale plus intensive et l'augmentation de la surveillance prénatale, ces grossesses restent à très haut risque. La collaboration du néphrologue avec les obstétriciens doit être continue avec une surveillance clinique et échographique mensuelle à partir du deuxième trimestre à la recherche d'un hydramnios et pour contrôle du développement fœtal. Par ailleurs, la formation d'un hydramnios lié à la diurèse osmotique fœtale in utero se révèle dans 50% des cas dans notre étude, et dans 55 à 70% des cas de la littérature. La ponction de liquide amniotique n'est presque jamais nécessaire (rarement mentionnée dans les publications, deux ponctions dans notre étude ont été réalisés), l'intensification de la dialyse suffit en général à atténuer ou à corriger ce phénomène [18]. Une menace d'accouchement prématuré, en relation avec la distension utérine liée à l'hydramnios ou avec d'autres phénomènes est rencontrée chez 75% des dialysées enceintes de la littérature, mais moins fréquemment dans nos observations environ 8%. Elle survient en moyenne à 28 SA et nécessite une tocolyse par béta-mimétiques ou inhibiteurs calciques, ainsi qu'une hospitalisation et un repos complet jusqu’à la naissance. L'accouchement a lieu par césarienne dans 16,6% des cas (2 patientes sur 12) pour nos patientes. Dans presque toutes les situations, la décision de césariser est prise dans l'urgence en raison de la survenue d'une complication maternelle ou fœtale. Un accouchement imminent, lorsqu'il peut être reconnu, amène à intensifier encore la dialyse dans les 48 heures qui le précèdent. L'objectif est que le bébé naisse avec une urémie et une créatininémie les plus basses possibles, afin de limiter les complications d'ordre osmotique à sa naissance. Il n'a été décrit aucune complication spécifique de ces accouchements de mères dialysées, notamment aucun problème hémorragique en dépit de l'administration d'HBPM lors des séances proches du terme. Dans notre expérience comme dans les autres séries, tous les enfants sont nés prématurément [5]. Si possible, la grossesse devrait être poursuivie jusqu’à 36 SA afin de diminuer les risques secondaires à la prématurité. L'attitude que nous avons observée est de décider du moment optimal de l'extraction au cas par cas [19]. Les résultats que nous rapportons nous amènent à penser qu'il existe également une place pour la grossesse chez les patientes en hémodialyse chronique. Même si cette grossesse en dialyse est techniquement possible, elle est également vivement déconseillée, l'idéal étant de pouvoir patienter jusqu’à la transplantation rénale, lorsque cette dernière est envisageable.

## Conclusion

Au total, la grossesse chez la dialysée est fortement déconseillée, car considérée comme à haut risque pour la mère et l'enfant, avec un faible taux de naissances vivantes (environ 40%). Mais sa survenue est le plus souvent inattendue par méconnaissance d'un retour de fertilité possible à la mise en dialyse. Son diagnostic est souvent tardif, et sa prise en charge technique est très lourde pour la patiente et pour l’équipe multidisciplinaire qui la suit. De plus, elle peut provoquer une hyperimmunisation maternelle préjudiciable à une future greffe rénale. De l’étroite collaboration et de la motivation des différents acteurs dépend aussi l'issue de la gestation. Aussi, est-il vivement conseillé à la femme dialysée d'attendre d'avoir été transplantée avec succès pour planifier une grossesse.
